# On Morphology of Aluminum–Gallium Nitride Layers Grown by Halide Vapor Phase Epitaxy: The Role of Total Reactants’ Pressure and Ammonia Flow Rate

**DOI:** 10.3390/ma17143446

**Published:** 2024-07-12

**Authors:** Arianna Jaroszynska, Michal Dabrowski, Petro Sadovy, Michal Bockowski, Robert Czernecki, Tomasz Sochacki

**Affiliations:** Institute of High Pressure Physics, Polish Academy of Sciences, Sokolowska 29/37, 01-142 Warsaw, Polandtsochacki@unipress.waw.pl (T.S.)

**Keywords:** nitride semiconductors, aluminum–gallium nitride (AlGaN), Halide Vapor Phase Epitaxy (HVPE), crystal growth, growth morphology, supersaturation

## Abstract

The focus of this study was the investigation of how the total pressure of reactants and ammonia flow rate influence the growth morphology of aluminum–gallium nitride layers crystallized by Halide Vapor Phase Epitaxy. It was established how these two critical parameters change the supersaturation levels of gallium and aluminum in the growth zone, and subsequently the morphology of the produced layers. A halide vapor phase epitaxy reactor built in-house was used, allowing for precise control over the growth conditions. Results demonstrate that both total pressure and ammonia flow rate significantly affect the nucleation and crystal growth processes which have an impact on the alloy composition, surface morphology and structural quality of aluminum–gallium nitride layers. Reducing the total pressure and adjusting the ammonia flow rate led to a notable enhancement in the homogeneity and crystallographic quality of the grown layers, along with increased aluminum incorporation. This research contributes to a deeper understanding of the growth mechanisms involved in the halide vapor phase epitaxy of aluminum–gallium nitride, and furthermore it suggests a trajectory for the optimization of growth parameters so as to obtain high-quality materials for advanced optoelectronic and electronic applications.

## 1. Introduction

Aluminum–gallium nitride (Al_x_Ga_1−x_N, where 0 < x < 1) combines the properties of gallium nitride (GaN) and aluminum nitride (AlN), thus creating a useful alloy for modern electronics and optoelectronics. Its application depends on the aluminum content. In optoelectronics, this alloy is crucial for the fabrication of quantum wells present in UV light devices like LEDs or lasers which find applications in surface disinfection, water purification, disease detection, material hardening, and counterfeit money detection [1,2,3,4,5,6,7]. It is said that a relaxed, free-standing Al_x_Ga_1−x_N substrate could enable the fabrication of continuous-wave (CW) operation of UV lasers and better performing UV light emitting diodes capable of operation at higher output powers through the combination of self-heating suppression and the reduction in series resistance [8]. As for the field of electronics, Al_x_Ga_1−x_N is used in high-voltage transistor designs which are important for more demanding, high-power applications [9,10,11]. It also plays a key role in improving the reliability of some semiconductor device designs by reducing strain in their epitaxial structures [12,13,14].

Regardless of the specific application, the use of the Al_x_Ga_1−x_N is limited to epitaxy carried out at the device structure building stage [15]. The semiconductor device designs based on the native Al_x_Ga_1−x_N substrate remain unexplored which originates from the fact that wafers with the desired Al composition are not available. This situation is due to difficulties in crystallizing bulk Al_x_Ga_1−x_N alloy which are fundamentally related to the thermodynamic stability of the two constituent nitrides: AlN and GaN. Aluminum nitride is thermodynamically stable at a high temperature at a nitrogen (N_2_) pressure of 1 atm. For GaN, thermodynamic stability is lost at 800 °C in the same pressure conditions. At higher temperatures, the equilibrium partial pressure of N_2_ becomes higher for GaN. Therefore, the growth of GaN at high temperatures (>800 °C) requires a high N_2_ pressure or a high nitrogen activity [16].

Nowadays, bulk GaN is predominantly crystallized on a large scale with the use of the halide vapor phase epitaxy (HVPE) method [17]. Meanwhile, AlN crystals are primarily obtained using the physical vapor transport (PVT) method. It should be noted that the crystallization of AlN by HVPE still exists only within the realm of laboratory research [18]. Applying the HVPE method for the crystallization of A_x_lGa_1−x_N appears to be challenging. Differences in the thermodynamic properties of both nitrides dictate the differences in conditions for their stable growth. Gallium nitride is crystallized using the HVPE method at a relatively low temperature (1000 °C) and in the excess of ammonia (NH_3_) to protect against its decomposition. Stable growth of HVPE-AlN occurs at a much higher temperature (1400 °C), which necessitates greater mobility of Al atoms on the seed surface and at significantly lower NH_3_ flows limiting the gas-phase reaction of the Al precursor with NH_3_. Combining these two systems for the simultaneous crystallization of AlN and GaN results in a situation where GaN loses its thermodynamic stability at high temperatures favorable for AlN crystallization. In these conditions, the desorption of Ga atoms from the seed surface begins to dominate over the adsorption of the Ga precursor. On the other hand, at low temperatures favorable for stable GaN crystallization, one can expect the dominance of Al precursor adsorption processes on the seed surface or even the reaction of the Al precursor with NH_3_ in the gas phase. The crystallization process of A_x_lGa_1−x_N is feasible from a thermodynamic perspective. First, thermodynamic analysis of crystallization using AlCl_3_, GaCl and NH_3_ as precursors was described by Koukitu et al. [19]. The presented calculations showed that a controllable growth of Al_x_Ga_1−x_N was possible using a low partial pressure of hydrogen (up to 10% of H_2_ diluted in the carrier gas, i.e., N_2_). Experimental confirmation of HVPE growth of A_x_lGa_1−x_N on sapphire seeds using an AlCl_3_–GaCl–NH_3_ system was also reported by Koukitu et al. [20]. Results confirmed that the alloy composition could be controlled in all ranges from GaN to AlN, only when a low partial pressure of H_2_ carrier gas was used. A typical growth rate was noted to be approximately 30 µm/h at 1100 °C. The morphology of the crystallized layers was not analyzed.

A supplement to the calculations described by Koukitu et al. [19] with a broader analysis of the influence of individual factors such as: temperature, the type of the carrier gas, the input partial pressures of NH_3_, and the Al and Ga precursors are discussed by Washiyama et al. [21]. In the referenced work, the supersaturation of Ga and Al during Al_x_Ga_1−x_N growth for given growth conditions was estimated. The calculations revealed that the Ga phase was close to the chemical equilibrium (supersaturation close to zero), while the Al supersaturation ratio was as high as 10^10^. Such a disparity in the supersaturation of reaction species can play a significant role in the stability of the growth of the ternary alloy. The calculations also showed that with low NH_3_ flow rates and two different diluent gases (H_2_ and N_2_), Al_x_Ga_1−x_N growth is in the thermodynamically limited growth regime at temperatures above 1040 °C. This implies that maintaining a constant ratio of Al and Ga precursors in the gas phase resulted in an observable increase in the Al content of the crystallized layers as the temperature rose. Several experimental groups sought to define a framework for process parameters that would allow the stable crystallization of Al_x_Ga_1−x_N with up to 30 at.% of Al content [22,23,24]. Most commonly, the growth temperature was set at 1100 °C so as to increase the mobility of Al atoms on the surface, therefore lowering the supersaturation for GaN crystallization. Layers with a thickness range of 1 to 20 µm were obtained. The use of a lower growth temperature was presented by Fujikura et al. [8]. In that work, the authors presented results which coincide with the thermodynamic calculations regarding the effect of the carrier gas on the relationship between the ratio of gaseous Al and Ga precursors and the final Al content of the crystallized layers. The authors used a growth temperature of 1050 °C for the crystallization Al_x_Ga_1−x_N.

This article provides an experimental supplement to thermodynamic calculations presented by Koukitu et al. and Washiyama et al. for of HVPE-Al_x_Ga_1−x_N crystallization [19,21]. The study focuses on presenting the impact of two growth parameters, NH_3_ flow rate and the reactants’ total pressure in the reactor, on the morphology of the Al_x_Ga_1−x_N growth. Additionally, the influence of these parameters on growth rates and Al content will also be commented on. The most optimal parameters for the growth of Al_x_Ga_1−x_N will be discussed along with a variety of problems encountered during research. The purpose of this work is to establish a framework of process parameters in which the post-growth morphology of Al_x_Ga_1−x_N layers is smooth and free of defects or other potential problems, while also retaining growth rate higher than Al_x_Ga_1−x_N grown by Metalorganic Vapor Phase Epitaxy (i.e., >1 µm/h). The state of morphology reflects the quality of crystal growth; therefore, problem-free morphology is the first step towards obtaining high-quality, free-standing Al_x_Ga_1−x_N crystals in the long-term.

## 2. Experimental Details

**Figure 1:**
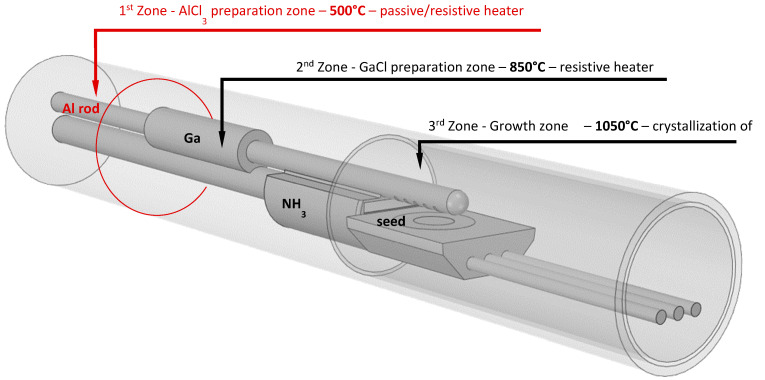
Illustration of the hot-wall quartz HVPE reactor with three temperature zones used in this work. The two primary reactants (AlCl_3_ and GaCl) are synthesized from Al and Ga metallic precursors in two independent zones. The reactants subsequently combine in the nozzle before the showerhead exit. Later, the mixture reacts with NH_3_ on the surface of the crystal seed.

For this work a quartz horizontal HVPE reactor (built in-house) with inserted quartz doping tubes was used (see Figure 1). The reactor is divided into three zones with varying temperatures. Solid cylindrical rods (6N purity, 10 cm length, 6 mm diameter) were used as the source of Al in the system. The Al rods were placed in the 1st Zone (temperature range 450–500 °C), where AlCl_3_ was synthetized according to the equation (Equation (1)). Liquid Ga was placed in the 2nd Zone, where GaCl was synthetized at 850 °C according to the equation (Equation (2)). Two key reactions occur in the 3rd Zone at 1050 °C, which ultimately result in the crystallization of Al_x_Ga_1−x_N according to the equation (Equations (3) and (4)) [20].

2Al(s) + 3Cl_2_(g) **→** 2AlCl_3_(g) (1)

2Ga(l) + Cl_2_(g) **→** 2GaCl(g) (2)

GaCl(g) + NH_3_(g) **→** GaN(s) + H_2_(g) + HCl(g) (3)

2AlCl_3_(g) + 2NH_3_(g) **→** 2AlN(s) + 3HCl(g) (4)

The research was conducted in two series (A and B), each consisting of five growth experiments. The parameters defining these experiments are two variables commonly occurring in the scientific literature related to crystallization of Al_x_Ga_1−x_N, the R parameter and the V/III ratio. In the physical sense, the R parameter represents how much aluminum compared to gallium is introduced into the system. It can be used to compare the concentration of aluminum in gas and solid phases. The R parameter and V/III ratios are defined by Equations (5) and (6), respectively.

(5) $$R=\frac{p^{0}\left(Al{Cl}_{3}\right)}{p^{0}\left(GaCl\right)+p^{0}\left(AlCl_{3}\right)}$$

(6) $$V/III=\frac{p^{0}\left(NH_{3}\right)}{p^{0}\left(GaCl\right)+p^{0}\left(AlCl_{3}\right)}$$

where the individual components were defined as: $p^{0}\left(GaCl\right)$—the input partial pressures of Ga precursor, $p^{0}\left(AlCl_{3}\right)$—the input partial pressures of Al precursor, and $p^{0}\left(NH_{3}\right)$—the input partial pressures of NH_3_.

The main parameter separating the series of experiments was the V/III ratio (A: V/III = 59, B: V/III = 21). The change in V/III ratio was mainly realized by regulating the NH_3_ flow; therefore, the article uses the concepts of decreasing (or increasing) V/III and decreasing (or increasing) NH_3_ flow interchangeably. In other words, within the context of this article, a decrease in NH_3_ flow results in a decrease of the V/III ratio and vice versa. Three experiments in each series were conducted at different total pressures within the reactor (800, 400 and 200 mbar). In experiments A4, A5, B4 and B5, the flow over either the Ga or Al precursors was shut off so as to analyze the crystallization of non-alloyed GaN and AlN in the system at 200 mbar. In addition to the former, non-alloyed GaN growth was tested in the system at 800 mbar (A0, B0) using two different V/III ratios as a reference for the other experiments. In order to maintain a constant V/III ratio, the flow of NH_3_ was reduced accordingly. The growth temperature, process time and N_2_, used as a carrier gas for all reagents, remained unchanged between the experiments. The defined growth process parameters are summarized in Table 1. Processes 1, 2 and 3 consisted of two growth stages. Initially, GaN layer was deposited for 15 min on a template composed of GaN grown by metalorganic vapor phase epitaxy on a sapphire substrate. Then, the precursor flow over metallic Al was turned on, and growth was conducted for 105 min. In the case of experiment 4 and 5 in both series, the total growth time lasted 120 min for GaN or AlN, respectively.

The morphology of the layers obtained from the processes described in Table 1 was characterized using Nikon Eclipse LV100ND Differential Interference Contrast (DIC) optical microscope (OM) with Nomarski contrast and both visible (VIS) and ultraviolet (UV) light. UV light was utilized for estimating the thickness of the deposited layers. The Al content of Al_x_Ga_1−x_N layers was determined using a Zeiss Ultra Plus scanning electron microscope (SEM) equipped with a Bruker Quantax400 Energy Dispersive Spectroscopy (EDS) module.

## 3. Results

**Figure 2:**
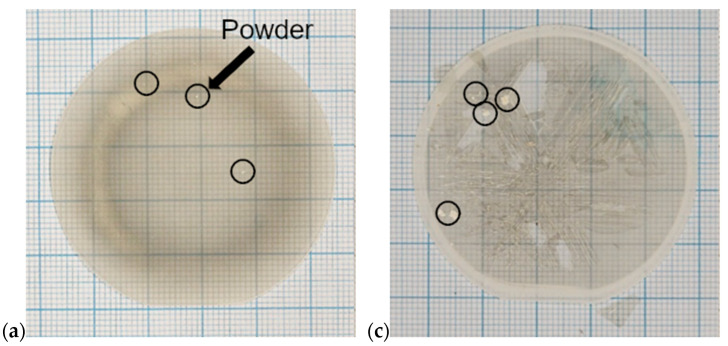

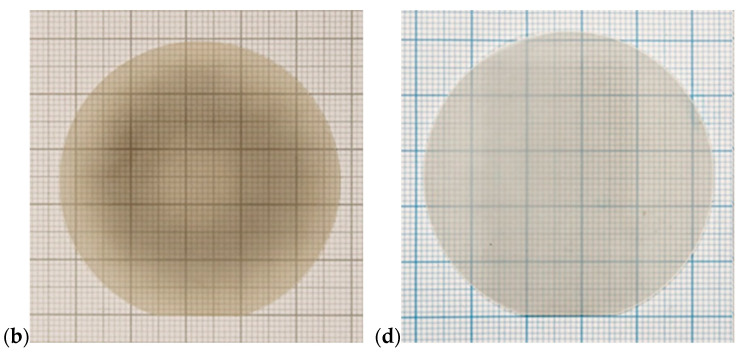
The top-down photographs of crystals grown in different growth conditions: (**a**) V/III = 59, *p* = 800 mbar, (**b**) V/III = 59, *p* = 200 mbar, (**c**) V/III = 21, *p* = 800 mbar, (**d**) V/III = 21, *p* = 200 mbar. Black circles mark the white powder observed for samples grown in the higher pressure.

Figure 2 contains the top-down photographs of crystals A1, A3, B1, B3 (Figure 2a, Figure 2c, and Figure 2d, respectively) which were grown according to the conditions outlined in Table 1.

**Figure 3:**
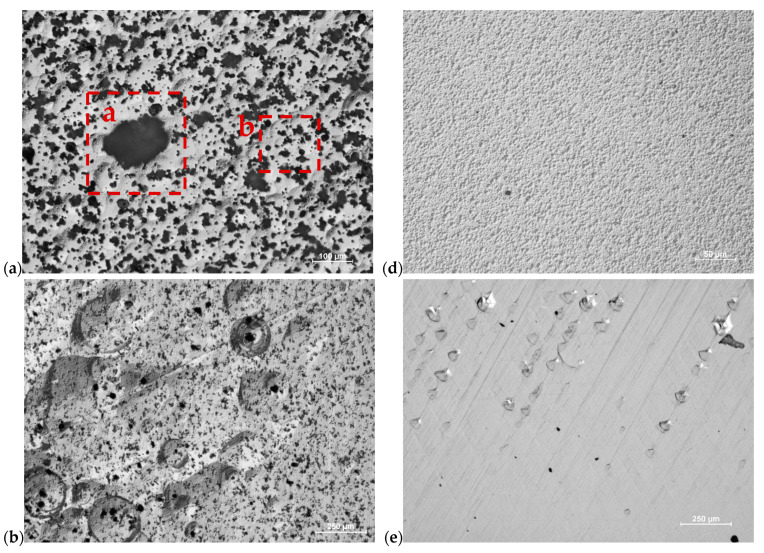

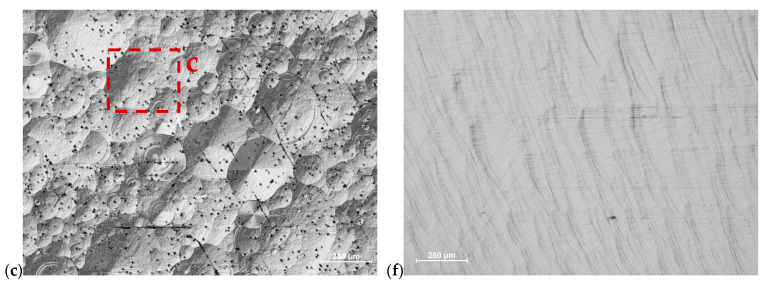
DIC images of the morphology for the as-grown crystals: (**a**) V/III = 59, *p* = 800 mbar, (**b**) V/III = 59, *p* = 400 mbar, (**c**) V/III = 59, *p* = 200 mbar, (**d**) V/III = 21, *p* = 800 mbar, (**e**) V/III = 21, *p* = 400 mbar, (**f**) V/III = 21, *p* = 200 mbar.

The cracking of the layer was observed for crystals grown in *p* > 200 mbar and V/III ratio of 21. An example of the cracking for the crystal grown in 800 mbar is shown in Figure 2c. Moreover, the white powder was observed on the surface of the crystals grown in pressures higher than 200 mbar regardless of the V/III ratio. Figure 2a,c contains black circles which mark places with the aforementioned powder observable on the surfaces of the crystals. The composition and origin of the white powder will be discussed in the following sections. The morphology of crystals A1, A2, A3, B1, B2 and B3 was characterized by a DIC microscope. The results are presented in Figure 3. The lowercase figure classifications correspond to the uppercase letters corresponding to a crystal (i.e., Figure 3a features crystal A1, etc.).

**Figure 4:**
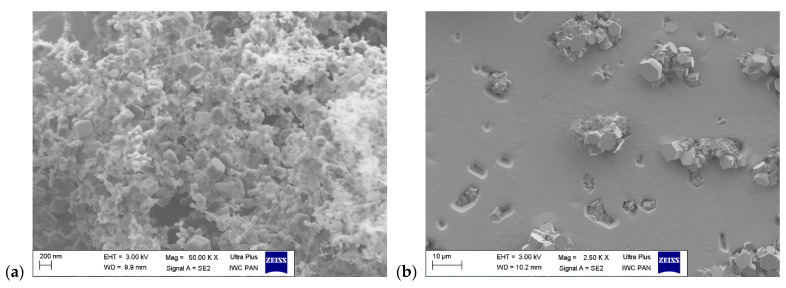

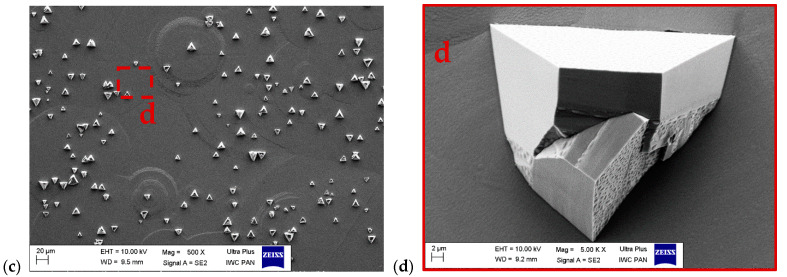
SEM images of the surface morphology of layers grown using V/III ratio = 59: (**a**) magnification of the object marked by rectangle a in Figure 3a, (**b**) magnification of continuous layer with black dots marked by rectangle b in Figure 3a, (**c**) magnification of the layer from rectangle c in Figure 3c, (**d**) higher magnification of the crystallite marked by rectangle “d” in (**c**).

Figure 3a–c present the evolution of morphology of grown layers in different pressures (Figure 3a: 800 mbar, Figure 3b: 400 mbar, Figure 3c: 200 mbar) and V/III ratio equal to 59. In these figures, continuous layers with multiple hillocks are observable. They are decorated by numerous black dots. Two types of black dots can be distinguished (marked by rectangles a and b; see Figure 3a). The dots enclosed in the rectangles a and b are presented again in Figure 4a and Figure 4b, respectively, where they are further investigated using a higher magnification by SEM. Black dots are the largest and most numerous for the layer grown in 800 mbar. The black dots become progressively smaller with the reduction in total system pressure (see Figure 3a,c). Both, size and quantity of these black dots are the smallest for the growth process conducted in the lowest pressure. Separately from the black dots, cracks are also present in the layer (see Figure 3b,c). Figure 3c contains a region of interest which is further investigated with SEM in Figure 4, just like the rectangles a and b previously presented in Figure 3a.

Figure 3d–f show the evolution of morphology in the A_x_lGa_1−x_N layers grown using V/III = 21 and pressures of 800, 400 and 200 mbar, respectively. The growth morphology shown in Figure 3d depicts a uniform, granular surface. Uniformity is retained with the reduction in pressure, although the morphology ceases to be granular (Figure 3e,f). The layer in Figure 3e is smooth, but the presence of cracks is noted. Small hillocks are present along the cracks. Lastly, Figure 3f contains a smooth, continuous layer with distinct macro-steps. Cracks are also present in the form of dark, intersecting lines. Figure 4 contains SEM images of the features marked in rectangles a, b and c described in the previous paragraph (see Figure 3a,c).

**Figure 5:**
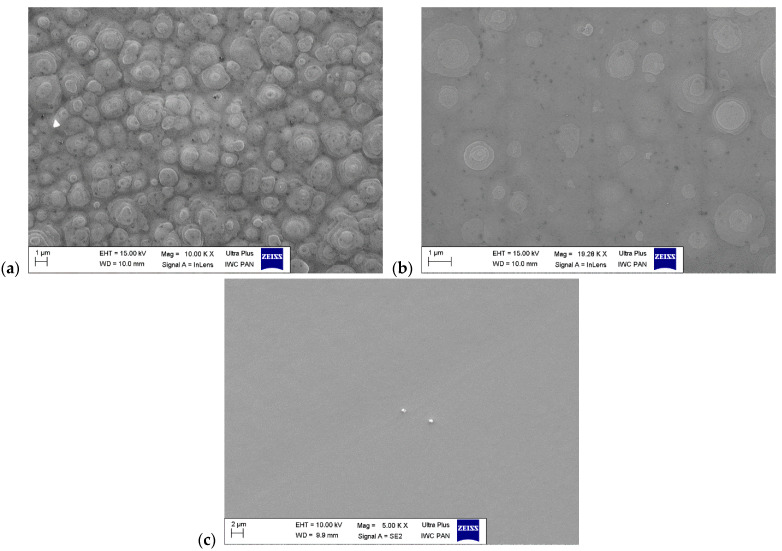
SEM images of the surface morphology of layers grown using V/III = 21: (**a**) magnification of granular surface presented in Figure 3d (800 mbar), (**b**) magnification of continuous layer presented in Figure 3e (400 mbar), (**c**) magnification of the layer presented in Figure 3f (200 mbar).

Figure 4a contains a closer view of the object enclosed in rectangle a in Figure 3a. A group of small, hexagonal crystallites are present. They are mutually connected, forming a large object with an irregular shape. In Figure 4b (rectangle b in Figure 3a), numerous crystallites with a regular hexagonal shape are observable. They appear to be embedded into the continuous layer. Figure 4c, which depicts rectangle c from Figure 3c, shows small triangular crystallites that are also partially inside in the crystal layer. These crystallites have well-defined facets (see Figure 4c) and are crystallographically oriented (see Figure 4a,b). SEM imaging was used for the detailed analysis of morphology in the layers in which the crystallites were absent. Figure 5 presents SEM images of these samples. 

Figure 5a shows a magnification of the granular morphology previously demonstrated in Figure 3d. Numerous growth hills are present on the surface of the sample. The layer grown in the reduced pressure of 400 mbar (Figure 5b) exhibits similar growth hillocks, although with a smaller population. The layer grown in 200 mbar is almost completely featureless. Its surface contained neither growth hills like in Figure 5a or Figure 5b, nor crystallites which are present in the samples grown in V/III = 59 (see Figure 4). Figure 5c depicts one of the very few regions on the sample where small imperfections are observable. The two imperfections in Figure 5c are likely to be crystallites.

Four supplementary experiments were performed in order to better understand how the growth of AlN and GaN proceeds in the system. In these experiments, non-alloyed AlN or GaN crystals were grown in two different pressures 800 mbar and 200 mbar (see: A4, A5, B4, B5 in Table 1). These crystals were later analyzed using DIC, OM and SEM.

**Figure 6:**
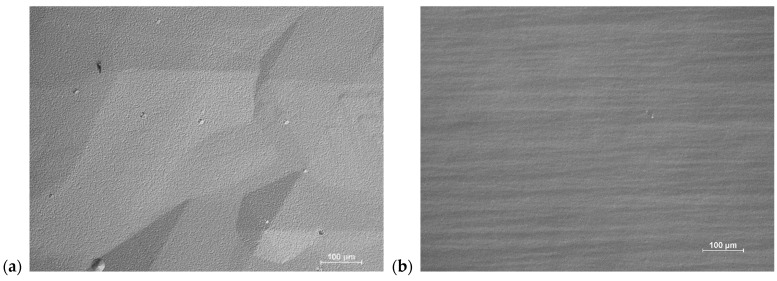

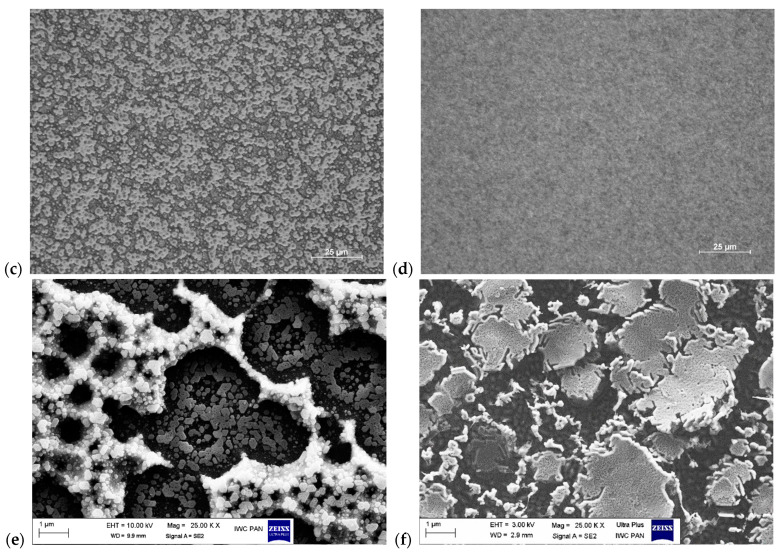
Morphology of the as-grown layers: (**a**) A4 (OM), visible growth hillocks, (**b**) B4—from (OM), visible macro-steps, (**c**) A5 (OM), visible grainy structure, (**d**) B5 (OM), visible rough surface (**e**) A5 (SEM), visible non-continuous layer with small flakes, (**f**) B5 (SEM), visible non-continuous layer with large flakes.

Figure 6a,b show the morphology of the GaN layer from processes conducted using high and low V/III ratios, respectively, at a pressure of 200 mbar, without Cl_2_ flow over the Al rod. Well-defined growth hillocks are visible in Figure 6a (V/III = 59). A significantly different morphology with well-developed macro-steps can be observed for the layer grown in V/III = 21 (see Figure 6b). Figure 6c,d show the morphology of the AlN layer from processes conducted using high and low V/III ratios, respectively, at a pressure of 200 mbar, without Cl_2_ flow over the liquid Ga. Figure 6c depicts a granular morphology, which is presented again in higher magnification as seen on SEM (Figure 6e). The morphology consists of a non-continuous, porous layer covered entirely with small hexagonal flakes. The flakes cover both the dark areas (depressions) and the bright areas (elevations). In contrast, the magnification of the morphology presented in Figure 6f reveals significantly larger and irregular flakes evenly distributed across the entire surface of the layer. Energy dispersive spectroscopy measurements confirmed that the layers crystallized in processes A4 and A5 are composed of pure GaN and the flakes which crystallized in experiments B4 and B5 constitute pure AlN. A summary of the results concerning the growth rates and Al content is presented in Table 2. The Al content refers to the atomic percentage within the continuous layer, not the individual crystallites.

Comparing the experiments in series A, a decrease in the growth rate was observed both after introducing AlCl_3_ into the system (A0 vs. A1) and after the reduction in the total reactor pressure (A0 vs. A4). In both cases, the decrease was 25% and 29%, respectively. The same comparison for the experiments in series B shows a similar decrease in the growth rate of 31% and 27%, respectively. A comparison of the growth rates of GaN layers from the A and B series (A0 vs. A3, B0 vs. B3) obtained from processes at high total pressure (800 mbar) against Al_x_Ga_1−x_N obtained from processes at low total pressure (200 mbar) indicated a comparable decrease in the average growth rate of 63% and 65%, respectively.

## 4. Discussion

The results describe two series of experiments which demonstrate the impact of the total pressure in the reactor and the NH_3_ flow rate in the growth zone on the morphology and Al content in the crystallized layers. Both parameters appear to have a significant influence on the supersaturation of Ga and Al in the system, impacting both the crystallization of GaN and AlN. Through the comparison of experiments A1, A2 and A3, an increase in the Al content in the crystallized layer is observed as the total pressure is reduced. Based on thermodynamic calculations, it can be stated that in the standard conditions for GaN crystallization (T = 1050 °C, high V/III = 59, *p* = 800 mbar), the supersaturation for AlN is extremely high as it was predicted by Washiyama et al. [21]. Furthermore, the diffusion of Al atoms on the seed surface is extremely limited. These theoretical considerations are reflected in this work, particularly when considering the case of the layers grown in the V/III ratio of 59. The analysis of morphology presented in Figure 3a (800 mbar), Figure 3b (400 mbar) and Figure 3c (200 mbar) suggests the formation of solid particles in the gas phase or direct nucleation of crystallites on the epitaxial layer. Similar observations were reported by Fujikura et al. [25]. The reason for the formation of these precipitates in the gas phase is still unknown. On the one hand, there is no thermodynamic basis for determining the presence of an AlN molecule in the gas phase. On the other hand, the process is carried out in the presence of NH_3_. This implies the possibility of the formation of stable solid complexes as a result of the reaction of AlCl_3_ and NH_3_ [26]. These complexes can be a source of nucleation in the gas phase. Then, formed particles can be the site of further nucleation and in the course of the process can lead to the development of small crystals, resulting in numerous Al_x_Ga_1−x_N crystallites visible on the surface in SEM (see Figure 4a) or even in the macroscopic scale (Figure 2a). Although, it should be noted that the reduction in pressure does reduce the quantity and size of the precipitates—suggesting that reducing pressure also reduces the formation of particles in the gas phase. Nevertheless, they were observed on all samples grown using V/III = 59. The crystallites likely fall on the surface during their formation, become embedded into the growing continuous layer, and then become new sources for further nucleation. This supposition is validated by the incoherent crystals which are partially submerged in the continuous layer shown in Figure 4b.

A reduction in the number of particles visible on the layer after growth (Figure 4a,b) and a decrease in their size (Figure 4b,c) is accompanied by a measurable increase in Al content in the continuous layer. The explanation for this phenomenon might be based on the fact that microparticles that form in certain growth conditions are more likely to consume the available AlCl_3_ in the system for their own expansion and consequently diminish the Al content in the growing continuous layer. With the lower pressure, and thus less formation of particles, the Al content in the layer from process A2 is higher than A1. A further increase in the Al content was observed in experiment A3, where the supersaturation for Al was further lowered to the point where formation of solid particles was effectively diminished. In experiment A3, the crystallites exhibit different features than the crystallites observed in processes A1 and A2. For one, they appear to have well-defined crystallographic orientation (Figure 4c). Presumably, the combination of low pressure (*p* = 200 mbar) and high V/III = 59 results in the AlN nucleation processes on the surface of the seed. One of the consequences of surface nucleation of AlN is the rapid growth nuclei as the growth process progresses.

A radical change in the growth morphology was observed when the V/III ratio was reduced, while the pressure was kept constant (experiments A1 and B1). In process B1, formation in the gas-phase was not observed. Instead, the growth proceeded on numerous hillocks of small size. As the pressure was reduced in the experiments with a lower V/III ratio = 21, the population of hillocks decreased while the Al content in the layer increased (experiment B2). This observation suggests that a significant portion of the AlCl_3_ precursor was consumed by the nucleation of AlN on the surface of the crystal grown as part of experiment B1. The formed nuclei could have become sources for the formation of growth hillocks, thus increasing their quantity. Further pressure reduction to 200 mbar (experiment B3) resulted in an Al_x_Ga_1−x_N layer, where not only were the crystallites absent but also no hillock formation was observed. The smooth surface obtained in the process B3 (V/III = 21 and *p* = 200 mbar) also exhibited the highest Al content and observable macro-steps.

High Al supersaturation observable for AlN crystallization at a relatively low temperature (1050 °C) is associated with the strong bonding of Al atoms to the surface and their low mobility on the surface. Moreover, the supersaturation for AlN under standard GaN growth conditions (process A1) is significantly higher than that for GaN. As proven by the analysis of growth morphology (process A1), a reduction in supersaturation is necessary. It is crucial to note that changing parameters affecting the Al supersaturation for the growth of AlN also impacts the Ga supersaturation for the growth of GaN. Therefore, determining the parameters in which the supersaturation for AlN is sufficiently low so that neither the formation and expansion of particles in the gas phase nor surface nucleation can occur while maintaining stable conditions for GaN crystallization is critical for the successful crystallization of Al_x_Ga_1−x_N with a smooth morphology.

Reducing the pressure from 800 to 400 mbar, while maintaining a high V/III ratio, does not cause significant changes in the growth rate of the crystallized layers (Table 2). In these conditions, the process is still conducted in an excess of NH_3_, and considering the thermodynamics involved in the growth, it is controlled by the transport of GaCl and AlCl_3_ to the surface of the growing layer. An increase in the Al content in the obtained layer was also observed. Therefore, it can be assumed that this change mainly results in the reduction in Al supersaturation, thus causing a reduction in particle formation in the gas phase. Further reduction in the pressure to 200 mbar results in the halving of the growth rate (from 18 to 9 µm/h), while also increasing the Al content in the obtained layer. This effect may be associated either with the reduction in the precursor’s contact time with the surface (due to higher gas velocities) or with the lowering of the equilibrium partial pressure of Ga or a combination of both of these effects. The shortening of contact time is associated with an order of magnitude increase in flow velocity with reactor pressure reduction from 800 to 200 mbar. The latter aspect is related to an increase in the desorption processes of Ga atoms from the surface. In these conditions, the rate at which Al atoms desorb from the surface also rises, although not as significantly as the desorption rate for Ga atoms. Consequently, it can be inferred that after reducing the pressure to 200 mbar, the surface nucleation of GaN slows down relative to the crystallization of AlN. For the latter, a transition from the nucleation regime to the crystal growth is observed.

The experimental results showed that reducing the NH_3_ flow (V/III: 59 → 21) did not significantly affect the growth rate neither in experiments focused on the growth of non-alloyed GaN (A0, A4 vs. B0, B4) nor Al_x_Ga_1−x_N (experiments A1, A2, A3 vs. B1, B2, B3) per the results shown in Table 2. Nonetheless, as the V/III ratio decreases the formation of particles in the gas phase and their expansion on the surface are suppressed. With the further reduction in pressure and lower V/III, the surface nucleation of AlN is also eliminated. It could be concluded that the reduction in the V/III ratio, or the NH_3_ flow in practical terms, shifts the growth conditions towards equilibrium conditions for A_x_lGa_1−x_N synthesis, albeit remaining distant from them.

It should be noted that lowering the pressure to 200 mbar is crucial for the reduction in Al supersaturation in the system. However, it should also be noted that reducing total pressure in the reactor without adjusting gas flows can limit the diffusion of reactants to the surface, ultimately halting growth or even leading to the decomposition of the substrate. Therefore, excessively lowering the pressure is not an advisable approach either. Consequently, reducing the input partial pressure of NH_3_, considering its excess relative to stoichiometric ratios, proved to be a prudent approach. A radical reduction in NH_3_ flow brings the process conditions closer to equilibrium conditions. This supposition is validated by the results from processes A4 and A5, as well as B4 and B5. The exclusion of AlCl_3_ from the system (process A4, Figure 6a and process B4, Figure 6b) results in the crystallization of a continuous GaN layer. It is important that in both cases (V/III = 59 and 21) the growth rates of the GaN layer doubled, when compared to the processes that also included the flow of AlCl_3_ (A3 vs. A4 and B3 vs. B4). Based on these observations, it can be assumed that the presence of AlCl_3_ (Al atoms on the seed surface) acts as a GaN growth inhibitor. In fact, this is further reflected in Table 2, where growth rates of experiments in which AlCl_3_ was absent (A0, B0, A4, B4) are higher than in their corresponding sister experiments (same V/III and pressure) where AlCl_3_ was present regardless of incorporated Al concentration. On the other hand, removing the GaCl flow from the system results in the co-occurring decomposition of GaN and the crystallization of small hexagonal flakes (experiment A5). In process B5, the decomposition of GaN is also observed, but the crystallization of AlN in the form of flakes of a larger size than those observed in A5 is seen. This indicates that merely reducing the input partial pressure of NH_3_ causes a shift in Al supersaturation, which shifts preferential growth from island based (Volmer–Weber) mode to layer-by-layer based (Frank–van der Merwe) mode.

A framework of process parameters with which a crystallite-free morphology with no hillocks and approximately 4 at.% aluminum content was obtained on a 18 μm thick Al_x_Ga_1−x_N layer. These results constitute an experimental foundation based on which further research aimed at obtaining thicker Al_x_Ga_1−x_N layers, and eventually free-standing Al_x_Ga_1−x_N, is planned. In this work, the main focus was on the detailed analysis of sample morphology using different total pressure of reactants and V/III ratios as variables. The presented results offer a new insight into Al_x_Ga_1−x_N growth morphology, which is rarely given attention in the scientific literature, using variables and growth conditions which are uncommon (e.g., total pressure of reactants as a variable, no admixture of H_2_ in N_2_ atmosphere). This research is complementary to calculation-based works and experimental works published in the scientific research, but not directly comparable to them. Nonetheless, some parallels with the existing published literature can be drawn. For example, the problems with parasitic nucleation are mirrored in ref. [8]. The authors do not elaborate on the composition of crystallites but note that the introduction of an enhanced etching effect by varying NH_3_ and H_2_ flows in the system has effectively suppressed the formation of crystallites. In this work, the suppression of crystallites was realized by lowering the total pressure of reactants and also the NH_3_ flow. Both solutions are fundamentally based on balancing the Al and Ga supersaturations so as to change the morphology of the growing crystals to prevent the formation of hillocks and crystallites on the surface. Our experimental results do not fully reflect the calculations-based works in refs. [19,20,21], which can be explained by, among other things, different growth conditions. These works served as an important reference for the choice of parameters which were investigated in this research. The presented effect of total pressure in the reactor and V/III ratio on supersaturation is in full agreement with the findings of authors in ref. [21]. The supersaturation was observed to decrease as either the pressure of reactants (p_Tot_ in ref. [21]) or the V/III ratio is reduced. References [19,20] suggest that the observed solid phase composition of aluminum should be higher than observed, resembling the value of the R parameter. The discrepancy can be explained by several factors, for example different reactor geometries and higher temperature in the growth zone (1100 °C [20]). However, the goal of this work was not to find process parameters in which aluminum content is the highest, but where the post-growth morphology is promising for further research aimed at crystallizing a free-standing Al_x_Ga_1−x_N crystal.

## 5. Summary

This study was conducted to understand how total pressure and NH_3_ flow rate (expressed as changes in V/III ratio) affect the growth characteristics and Al content of crystallized Al_x_Ga_1−x_N layers. The results revealed that both the total pressure and NH_3_ flow rate have significant effects on the supersaturation levels governing AlN and GaN crystallization, which translated into profound changes in morphology and Al incorporation. It was shown that lowering the total pressure increased the Al content of the crystallized layers, due to diminished formation of particles in the gas phase and their expansion. This was proven by changes occurring in the morphology of the layers, with the reduced pressure that led to fewer and smaller incoherent crystallites embedded in the continuous layers and an increase in its Al content. A reduction in the V/III ratio produced a radical change in the growth morphology for the samples grown in *p* = 800 mbar. The morphology transformed from being covered by crystallites formed in the process of gas-phase nucleation growth, to a hillock-dominated surface for the growth in 400 mbar. A completely smooth layer was obtained by a further reduction in the total pressure to 200 mbar. This suggests that at lower pressures AlCl_3_ is mainly consumed in the surface crystallization processes of A_x_lGa_1−x_N, which led to smoother layers with a higher Al content. The experiments also highlighted the delicate balance between maintaining conditions favorable for GaN growth while reducing the Al supersaturation so as to prevent the formation of particles in the gas phase and promote layer by layer growth. Lowered pressure and adjusted NH_3_ flow were the key components to achieve this balance. The reduction in pressure to 200 mbar proved to be particularly effective for the successful crystallization of AlN, without a significant negative impact on GaN growth. In the end, Al_x_Ga_1−x_N layers featuring macro-steps on the surface, approximately 4% Al content (x = 0.04) and a thickness of 18 μm, were successfully crystallized as part of this work.

## Figures and Tables

**Table 1:** The summary of growth processes with their defining parameters (pressure, V/III ratio and the R parameter).

Experiment	Pressure [mbar]	V/III Ratio	R
A0	800	59	0
A1	800	59	0.3
A2	400	59	0.3
A3	200	59	0.3
A4	200	59	0
A5	200	59	1
B0	800	21	0
B1	800	21	0.3
B2	400	21	0.3
B3	200	21	0.3
B4	200	21	0
B5	200	21	1

**Table 2:** Results of estimated growth rates and Al content measurements in crystalized Al_x_Ga_1−x_N layers from growth processes 1 to 3 and GaN and AlN layers from follow-up experiments 4 and 5, respectively.

Experiment	Layer Thickness [μm]	Growth Rate [μm/h]	Al Content [%]	Pressure [mbar]	V/III Ratio	R
A0	47.7	24	0.00	800	59	0
A1	36.4	18	0.00	800	59	0.3
A2	38.7	19	1.48	400	59	0.3
A3	17.1	9	3.37	200	59	0.3
A4	34.5	17	0.00	200	59	0
A5	-	-	50.00	200	59	1
B0	51.5	26	0.00	800	21	0
B1	36.8	18	0.66	800	21	0.3
B2	30.7	15	1.87	400	21	0.3
B3	18.4	9	4.08	200	21	0.3
B4	38.9	19	0.00	200	21	0
B5	-	-	50.00	200	21	1

## Data Availability

Data are contained within the article.
